# Design and characterization of mesoscopic dielectric cuboid antenna for operation in WR-3.4 waveguide bandwidth (220–330 GHz)

**DOI:** 10.1038/s41598-023-42640-x

**Published:** 2023-09-20

**Authors:** Towa Ohno, Ayumu Yabuki, Keizo Inagaki, Atsushi Kanno, Junichi Nakajima, Norihiko Sekine, Shintaro Hisatake

**Affiliations:** 1 Gifu University, Gifu, 501-1193 Japan; 2 SoftBank Corp., Tokyo, 105-7529 Japan; 3 National Institute of Information and Communications Technology, Koganei, 184-8795 Japan; 4 Nagoya Institute of Technology, Nagoya, 466-8555 Japan

**Keywords:** Electrical and electronic engineering, Electronics, photonics and device physics

## Abstract

We designed a mesoscopic dielectric cuboid antenna connected to a flangeless WR-3.4 open-ended waveguide, and the antenna characteristics at 300 GHz were examined through simulations and experiments. Simulations confirmed that the flangeless design eliminated the flange-induced ripples in the radiation pattern, whose shape varied with frequency, and that the antenna operated in the full bandwidth of the WR-3.4 waveguide (220–330 GHz). Prototypes were then fabricated based on the simulation findings. A prototype with an antenna aperture area of 1.5 mm $\times$ 1.5 mm and an antenna length of 2.35 mm exhibited an antenna gain of 17.2 dBi at 300 GHz and a voltage standing wave ratio of less than 1.5 throughout the WR-3.4 waveguide bandwidth. The level of the side lobes at about $\pm 30$ degrees in the E-plane pattern was approximately $-10$ dB that of the main lobe. Therefore, the proposed antenna, connected to a flangeless waveguide, is a promising antenna for use in future short-range high-speed terahertz wireless applications such as kiosk downloads and board-to-board communication.

## Introduction

Because of its wide frequency bandwidth, the terahertz band is being considered not only for the backhaul and fronthaul of future high-speed, high-capacity mobile communication systems (beyond 5G/6G)^1^ but also for various short-range applications, such as kiosk downloads and board-to-board communication^2^. In particular, the 300 GHz band is being studied and developed actively worldwide. The development of high-gain antennas is important for 300 GHz wireless communication applications because of the proportional increase in free-space propagation loss with the square of frequency. In general, a trade-off exists between the physical aperture area and gain of an antenna; smaller antennas have lower gains. Therefore, achieving high-speed wireless communication in the 300 GHz band for mobile terminals requires a balance between antenna size and gain, so compact high-gain antennas should be designed.

Various types of compact antennas operating in the 300 GHz band have been proposed^3–13^. A step-corrugated horn antenna with dimensions of 3.2 mm $\times$ 2.8 mm $\times$ 2.8 mm and a gain of 18 dBi was demonstrated in a past study^3^. Dielectric resonator antennas (DRAs) made of high-dielectric-constant materials for the 300 GHz band have been investigated, and they are drawing attention as alternative on-chip options to planar antennas^9–11,14–18^, although antennas with resonant structures exhibit narrowband operation around their resonance frequencies. An approach to achieving wideband DRA operation was proposed where multiple higher-order dielectric resonator modes and cavity mode are simultaneously excited; the peak antenna gain was 8.6 dBi at 290 GHz, with a 3-dB gain bandwidth of 55 GHz (270–325 GHz)^10^. However, even with such frequency bandwidth extension, the potential operating bandwidth limitation due to the resonant structure of DRAs may hinder the complete utilization of the extensive frequency bandwidth of the terahertz band. A dielectric rod waveguide antenna (DRWA) is a surface-wave antenna that operates without using resonance. It has good directivity in the end-fire direction, and its characteristics have been studied analytically and experimentally, including those in the millimeter-wave and terahertz bands^12,13,19–30^. The maximum gain and minimum half-power beamwidth (HPBW) can be achieved by DRWAs by following Zucker’s design rules^27^. However, the length of the rod must be several tens of wavelengths to obtain sufficient gain for short-range communication, which requires considerable space when mounting such an antenna on a device. In^12^, a DRWA with a 20 mm radiation taper was verified; the $\vert \hbox {S}_{11}\vert$ was below $-10$ dB throughout the 75–1000 GHz band. In^29^, a DRWA with a 16 dBi gain and $\vert \hbox {S}_{11}\vert$ $< -15\,\hbox {dB}$ band from 75 to 110 GHz was one of the antenna elements constituting an antenna array for the millimeter-wave band.

Recently, we developed and proposed a mesoscopic dielectric cuboid antenna (DCA) and demonstrated its operation experimentally^31,32^. A DCA controls the wavefront using a wavelength-scale dielectric cube. Inherent broadband operation is possible because this antenna does not use resonance. We also demonstrated 17.5 Gbps wireless transmission in the 300 GHz band using a DCA sized 1.2 mm $\times$ 1.2 mm $\times$ 1.36 mm; the maximum antenna gain was approximately 15 dBi at 300 GHz^32^. However, these DCAs fabricated and demonstrated in the 300 GHz band were designed with waveguide flanges behind them, which caused ripples in the radiation pattern, especially in the E-plane. Moreover, the maximum gain direction varied with the operation frequency, which degraded the frequency characteristics of the antenna gain in a certain radiation direction. This is a critical problem for terahertz wireless communication systems using wide frequency bands based on frequency multiplexing methods, such as orthogonal frequency division multiplexing (OFDM).

This paper investigates the effect of cuboid dimensions on the characteristics of a DCA connected to a flangeless WR-3.4 waveguide (i.e., no metal plate is placed just behind the DCA). Simulations show that the flangeless design eliminates the ripples in the radiation pattern. The maximum antenna gain is reduced, even though the gain degradation can be recovered by slightly extending the antenna length. We also find an optimal antenna length for a given antenna aperture area. This optimal length resulted in the maximum antenna gain and a radiation pattern with a high rotational symmetry of the main lobe and a low side-lobe level (SLL). Based on the simulation results, we fabricated three DCA prototypes with different dimensions and measured their basic characteristics, namely, their antenna gains, radiation patterns, and $\vert \hbox {S}_{11}\vert$. A DCA prototype sized 1.5 mm $\times$ 1.5 mm $\times$ 2.35 mm provided a gain of 17.3 dBi at 300 GHz, and both the 3-dB gain bandwidth and $\vert \hbox {S}_{11}\vert$ $<-14.0$ dB bandwidth, corresponding to the bandwidth in which the voltage standing wave ratio (VSWR) is less than 1.5, covered the entire WR-3.4 waveguide bandwidth (220–330 GHz). Thus, the proposed DCA, which does not have a metal plate (waveguide flange), is a promising antenna for use in future short-range high-speed terahertz wireless applications such as kiosk downloads and board-to-board communication.

## Effects of waveguide flange

**Figure 1:**
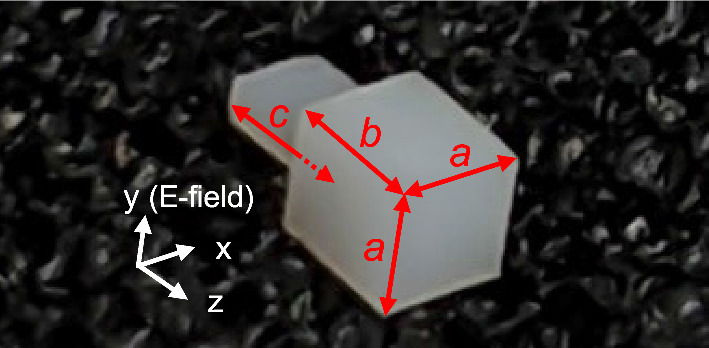
Photo of the DCA.

The proposed DCA for the 300 GHz band has a cuboidal radiating part ($a \times a \times b$) and a protruding connecting part (length *c*), as shown in Fig. 1. The cross-sectional dimensions of the connecting part are identical to the inner wall dimensions of a rectangular waveguide (0.864 mm $\times$ 0.432 mm WR-3.4 waveguide), and the DCA is inserted directly into the waveguide. The DCA material is polytetrafluoroethylene (PTFE), a low-loss dielectric at 300 GHz. PTFE has a relative permittivity of 2.038 and a loss tangent of 0.0011 at 300 GHz^33^. The phase velocity of an electromagnetic wave in a dielectric is slower than that in free space; therefore, the shape of the wavefront can be changed to generate a photonic jet^34–36^. This effect enables the conversion of the spherical waves radiated by the waveguide into planar waves to realize high antenna gains.

**Figure 2:**
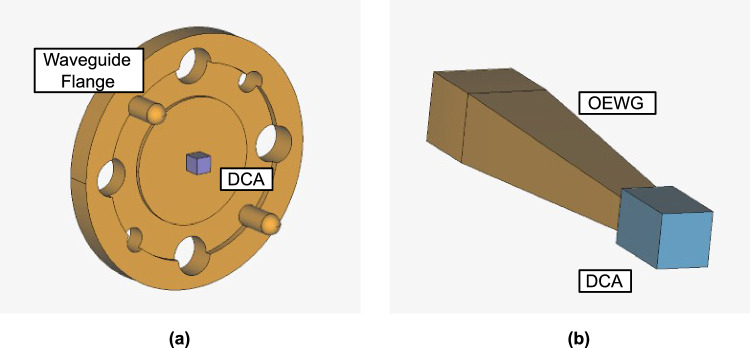
Simulation models of DCA and waveguide; (**a**) flanged waveguide and (**b**) open-ended waveguide (OEWG).

Through simulations, we examine the effect of the waveguide flange on the radiation patterns and gains of the DCA. Fig. 2 shows the simulation models of the DCA loaded on the waveguide with (Fig. 2a) and without (Fig. 2b) the waveguide flange. We use an open-ended waveguide (OEWG) model. The material of the waveguide is perfect electrical conductor. The waveguide flange (UG387) has a diameter of 19.05 mm and is significantly larger than the DCA. In the flangeless scenario, the DCA is loaded on the OEWG.

**Figure 3:**
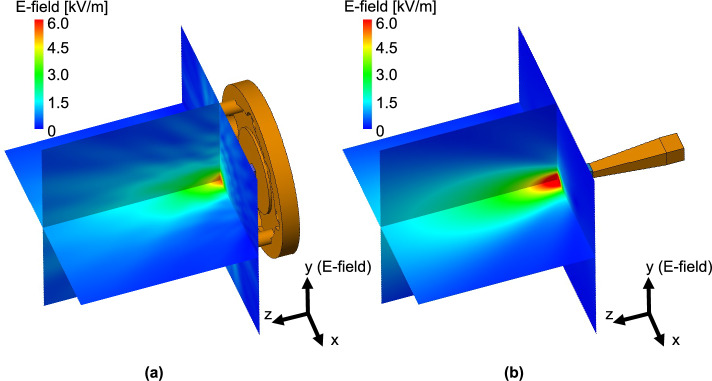
Simulated electric field distribution around the DCA with flanged waveguide and OEWG at 300 GHz, (**a**) with flanged waveguide and (**b**) with OEWG.

**Figure 4:**
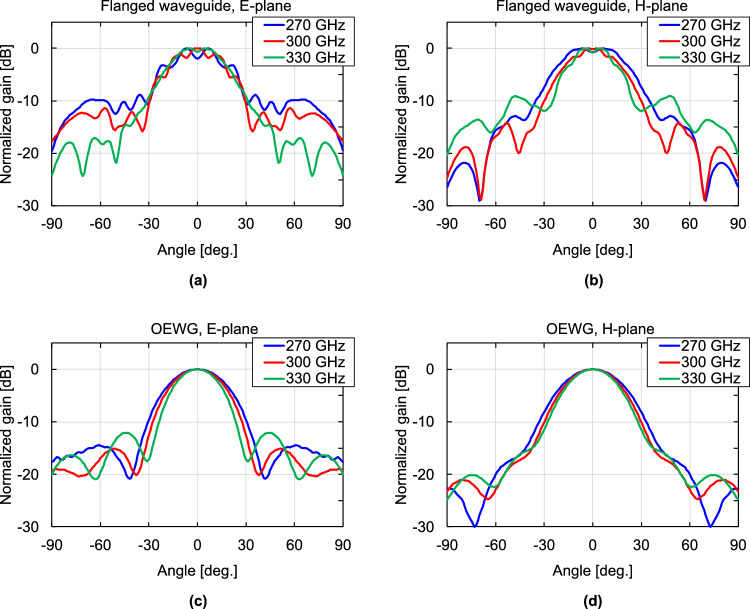
Simulated radiation patterns of DCA with flanged waveguide and OEWG, (**a**) E-plane pattern with flanged waveguide, (**b**) H-plane pattern with flanged waveguide, (**c**) E-plane pattern with OEWG, (**d**) H-plane pattern with OEWG.

**Figure 5:**
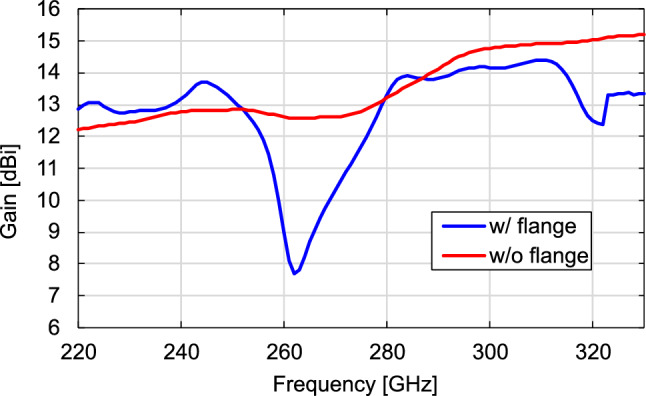
Comparison of simulated gains in forward direction with flanged waveguide and OEWG.

Fig. 3 depicts the simulation results of the electric field distribution around the DCA with and without the waveguide flange at 300 GHz. As shown in Fig. 3a, radiation from the waveguide flange is observed and it interferes with the radiation from the DCA. The interference causes ripples in the radiation pattern and the direction of maximum gain varies, affecting the frequency response of the forward gain. Figure 4 shows the normalized radiation patterns of the DCA with and without the waveguide flange obtained in the simulation. The radiation patterns with the waveguide flange exhibit ripples in the main lobes. The shapes of the ripples and the positions of the gain peaks vary with frequency. Figure 5 shows the frequency characteristic of the gain obtained in the simulation at a radiation angle of 0 degrees (forward gain) with and without the waveguide flange. As shown in Fig. 5, the presence of the waveguide flange enhances the gain, however the radiation pattern distortion leads to a variation in the forward gain with frequency. In this example, the gains degrade by approximately 5 and 2 dB at 262 and 320 GHz, respectively. Transmission demonstrations have been conducted previously for the direction of the maximum antenna gain at 300 GHz with 17.5 Gbps, using relatively narrow bandwidth^32^. However, such abrupt gain reduction is a serious problem for terahertz wireless communication systems that use a wide frequency band based on frequency multiplexing schemes, such as OFDM. Moreover, when the OEWG is used, i.e., the DCA is loaded on the flangeless waveguide, the forward gain generally increases consistently with frequency, and a 3-dB gain bandwidth is maintained throughout the frequency range (220–330 GHz). These results suggest that the waveguide flange significantly affects the radiation characteristics of the DCA and the use of an OEWG can reduce this impact.

## Design of flangeless DCA

In this section, we show the dependence of the gain and radiation pattern of the DCA on its physical dimensions and discuss the radiation efficiency of the DCA based on the simulations. Moreover, we examine the effect of applying a matching taper to the connection between the DCA and the waveguide and optimize this matching taper to realize WR-3.4 full-band operation (220–330 GHz).

### Dependence of DCA gain and radiation pattern on physical dimensions

**Figure 6:**
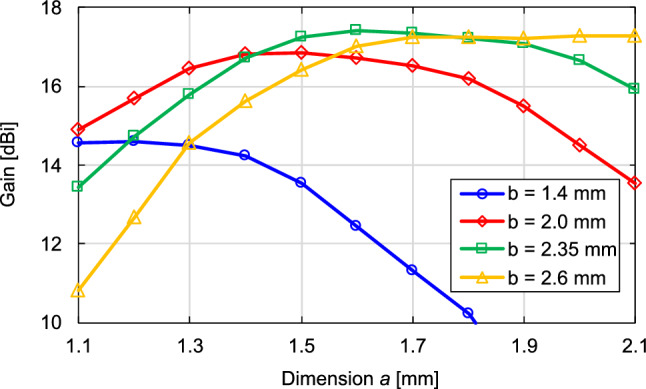
Simulated gain for the dimensions of the cuboid at 300 GHz.

First, we examine the dependence of the antenna gain and radiation pattern of the DCA on its physical dimensions at 300 GHz. For simplicity, we use a simplified OEWG model, just a straight waveguide with a wall thickness of 0.1 mm. The DCA has three dimensional parameters: the lengths of the cuboid (*a* and *b*) and the length of the connecting part (*c*). Fig. 6 presents the simulated characteristics of the gain with *a* at various *b*. We set $c =$ 1 mm in these simulations. As shown in Fig. 6, both *a* and *b* affect the gain, and an optimal dimension is observed from the perspective of the gain. For example, when $b =$ 1.4 mm, a gain of 14.6 dBi is achieved at $a =$ 1.2 mm. However, increasing *a* beyond this value decreases the gain instead of further improving it. As the value of *b* increases, the value of *a* that gives the maximum gain for a given *b* also increases, indicating that increasing the DCA gain requires a larger antenna volume. When $b =$ 2.35 mm, a maximum gain of 17.4 dBi is obtained at $a =$ 1.6 mm. However, even at $b =$ 2.6 mm (a 40$\%$ increase in antenna volume), the maximum gain remains approximately 17.4 dBi at $a =$ 1.8 mm. Thus, setting $a =$ 1.5 mm for the DCA is a good guideline from the perspective of achieving high gains and a compact size at 300 GHz.

**Figure 7:**
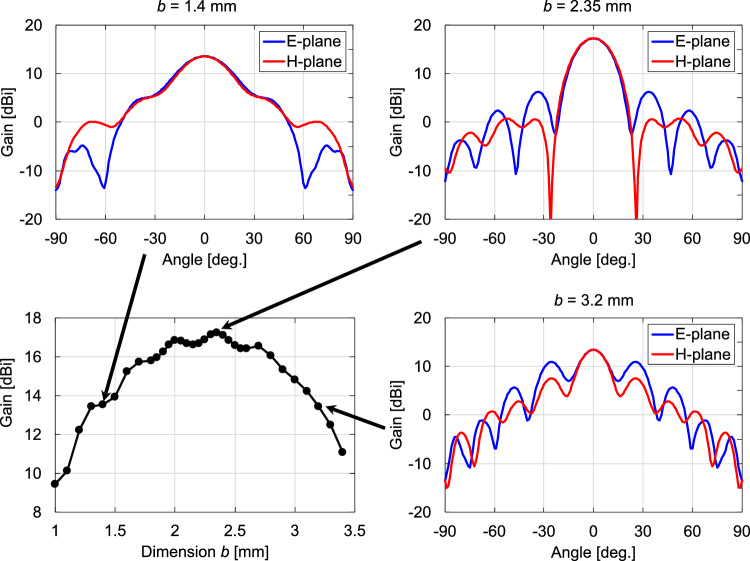
Variations in gain and radiation pattern with respect to *b* at *a* = 1.5 mm at 300 GHz.

**Figure 8:**
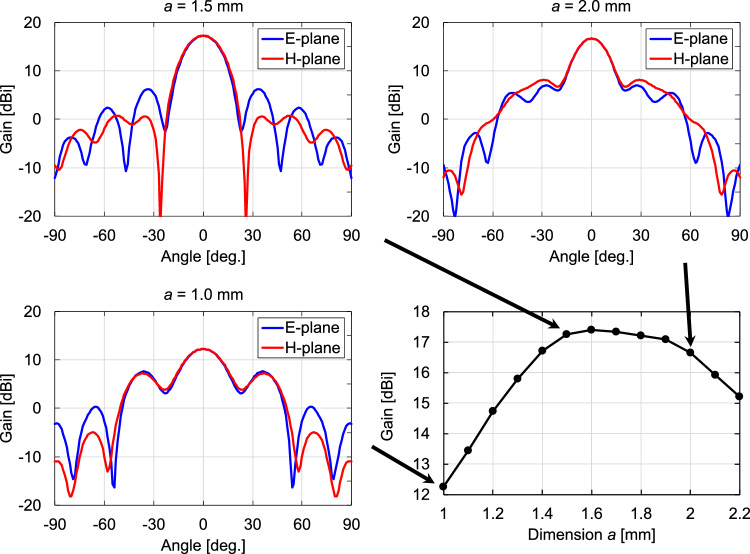
Variations in gain and radiation pattern with respect to *a* at *b* = 2.35 mm at 300 GHz.

Next, we examine the gain and radiation pattern by varying *b* at a fixed $a =$ 1.5 mm. Fig. 7 shows the simulated gains and radiation patterns for the representative dimensions as *b* varies from 1 to 3.4 mm. As *b* increases from 1 mm, the gain accordingly increases, peaking at approximately $b =$ 2.35 mm. The gain decreases as *b* further increases. The DCA works as a relatively high-gain antenna by converting the spherical wavefront into a planar shape. The principle of the operation is based on the phase velocity difference of the waves between the inside and outside of the DCA. The rate of wavefront conversion per unit propagation distance depends on the width *a* of the DCA. Therefore, the optimal value of *b* is the length that gives the propagation distance at which the wavefront is flattest at the aperture end (i.e., has the highest gain) for a given *a*. The radiation patterns of both the E- and H-planes have shoulders at approximately $\pm 40$ degrees. In addition, the E-plane characteristics show dips at approximately $\pm 60$ degrees. As *b* increases from 1.4 mm, the gain increases, and the position of the side lobe in the E-plane pattern becomes closer to the center, resulting in unimodal main-lobe patterns in both the E- and H-planes. At $b =$ 2.35 mm, where the maximum gain is achieved within the dimension *b* range, the main lobes in the E- and H-plane patterns are almost identical; i.e., the radiation patterns have high rotational symmetry. In the E- and H-plane patterns, the first side lobes appear at 33 and 35 degrees from the main lobes, with SLLs of $-11$ and $-16.5$ dB, respectively. The HPBW is 21.4 degrees in the E-plane and 22.3 degrees in the H-plane. As *b* increases from 2.35 mm, the SLLs increase. As shown in Fig. 7, at $b =$ 3.2 mm, a relatively large side lobe with an SLL of −2.7 dB appears at ±25 degrees in the E-plane pattern. Fig. 8 shows the relationship between the antenna dimensions and antenna gain as *a* varies from 1 to 2.2 mm when *b* is fixed at 2.35 mm. The radiation patterns for the representative dimensions are also shown. For both small ($a =$ 1 mm) and large ($a =$ 3.2 mm) dimensions, the SLLs increase. below −10 dB at $a =$ 1.5 mm, where the gain is at its maximum. Furthermore, at $a =$ 1.5 mm, the main-lobe characteristics of the E- and H-plane patterns are almost identical. The results in Figs. 7 and 8 indicate that the use of a DCA with $a =$ 1.5 mm and $b =$ 2.35 mm achieves a symmetric main-lobe pattern and a high antenna gain at 300 GHz.

### Frequency characteristics of radiation efficiency and $\vert \hbox {S}_{11}\vert$

**Figure 9:**
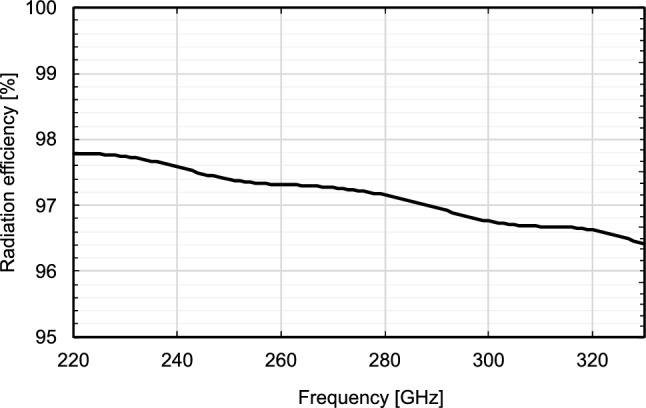
Simulated radiation efficiency at $a =$ 1.5 mm, $b =$ 2.35 mm.

Figure 9 shows the calculated frequency characteristics of the radiation efficiency at $a =$ 1.5 mm, $b =$ 2.35 mm, and $c =$ 1 mm. The total length of the dielectric part of the DCA is *b* $+$ $c =$ 2.35 mm $+$ 1 mm $=$ 3.35 mm. Although the radiation efficiency decreases with an increase in frequency, it remains higher than 96$\%$ throughout the WR-3.4 waveguide bandwidth (220–330 GHz). At 300 GHz, the radiation efficiency is 96.8$\%$. Please note that the radiation efficiency depends on the dielectric loss of the PTFE.

**Figure 10:**
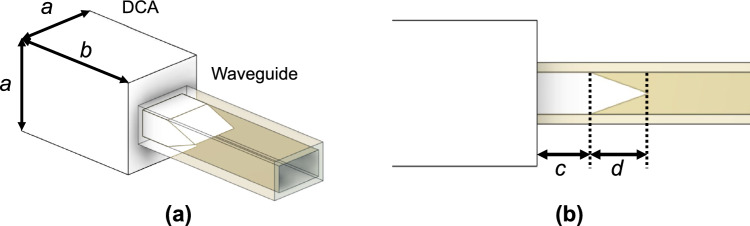
Schematic of DCA with matching taper, (**a**) 3D view, (**b**)side view.

**Figure 11:**
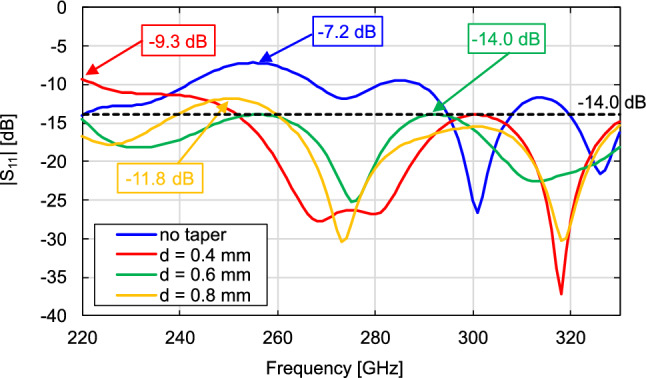
Effect of changes in length of matching taper *d* on simulated $\vert \hbox {S}_{11}\vert$ of DCA.

Finally, we examine the frequency characteristic of $\vert \hbox {S}_{11}\vert$ of the DCA. To decrease the impedance mismatch, we add a matching taper to the protruding connecting part, as shown in Fig. 10. The matching taper reduces reflections by gradually changing the characteristic impedance at the connecting point between the waveguide and antenna^37^. We confirm that the characteristics identified in the previous section are hardly affected by the presence or absence of the matching taper. Figure 11 shows the $\vert \hbox {S}_{11}\vert$ characteristics from the simulations without the matching taper (taper length $d =$ 0 mm) and at $d =$ 0.4, 0.6, 0.8 mm. The boxed numbers in the figure indicate the maximum values of $\vert \hbox {S}_{11}\vert$ for each *d*. The dimensions of the radiating section ($a =$ 1.5 mm and $b =$ 2.35 mm) are determined based on previous investigations. We set *c* to 0.55 mm, considering that varying *c* does not significantly affect the antenna characteristics; however, excessively short lengths may be inadequate for supporting the DCA, and excessively long lengths may increase dielectric losses and reduce radiation efficiency. The protruding connecting part (length: *c*) works simply as a waveguide filled with dielectric and does not play a significant role in return loss reduction. Without the matching taper, $\vert \hbox {S}_{11}\vert$ exceeds −10 dB in the ranges of 242–267 and 281–288 GHz. At $d =$ 0.6 mm and $d =$ 0.8 mm, the $\vert \hbox {S}_{11}\vert$ values are below −10 dB throughout the WR-3.4 waveguide bandwidth, confirming full-band matching. Among these lengths, $d =$ 0.6 mm achieves the lowest maximum $\vert \hbox {S}_{11}\vert$ value of −14.0 dB, which corresponds to the VSWR of less than 1.5, throughout the bandwidth.

## Prototyping and measurement

**Figure 12:**
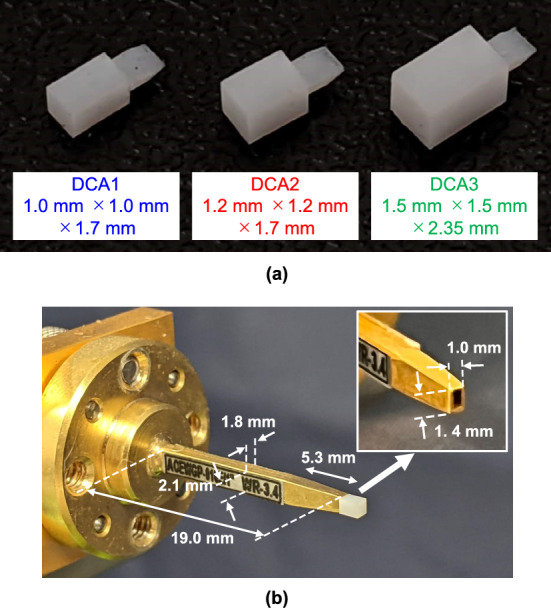
Photographs of fabricated prototyping antennas and OEWG. (**a**) Prototypes and (**b**) OEWG (Elmika: AOEWGP-010E/1).

We fabricated three prototypes and measured the frequency characteristics of the antenna gain and $\vert \hbox {S}_{11}\vert$. We also measured the radiation patterns of the DCA at $a =$ 1.5 mm and $b =$ 2.35 mm. Fig. 12a is a photo of the fabricated DCAs. All prototypes were made of PTFE and fabricated through machining. A matching taper with $d =$ 0.6 mm and $c =$ 0.55 mm was attached to all prototypes. The DCA dimensions, determined based on the simulations, were as follows:

- DCA1 was designed to obtain approximately the same antenna gain as that in^32^ ($a =$ 1.2 mm and $b =$ 1.36 mm) with a waveguide flange. Based on the simulations, the DCA dimensions were set to $a =$ 1 mm and $b =$ 1.7 mm. Although the reduction in antenna gain due to the absence of the waveguide flange was compensated for by lengthening *b*, the antenna volume reduced from 1.96 to 1.70 mm$^{3}$ (an approximately 13$\%$ reduction).
- DCA2 was designed to obtain the maximum antenna gain with the same aperture area ($a =$ 1.2 mm) as in^32^ with a waveguide flange. As seen in Fig. 7, a peak appeared in the gain-versus-*b* characteristic. Based on the simulations, *b* was set to 1.7 mm for the maximum gain at $a =$ 1.2 mm.
- DCA3 was designed to achieve higher gains while maintaining a compact antenna size. We set $a =$ 1.5 mm and $b =$ 2.35 mm, as discussed in the previous section.

**Figure 13:**
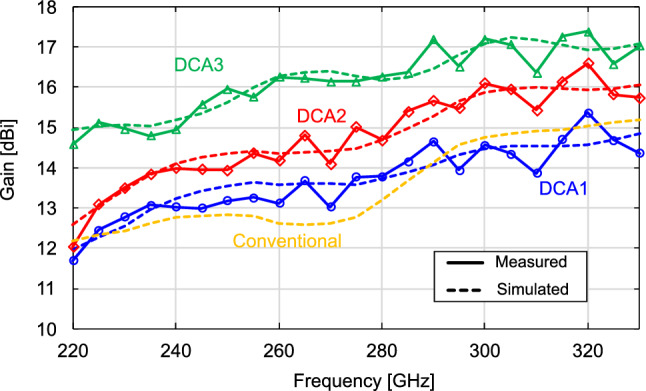
Frequency characteristics of measured and simulated gains.

**Figure 14:**
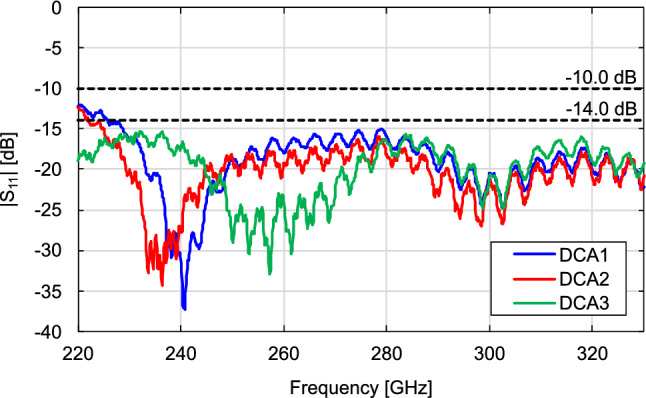
Frequency characteristics of measured $\vert \hbox {S}_{11}\vert$.

**Figure 15:**
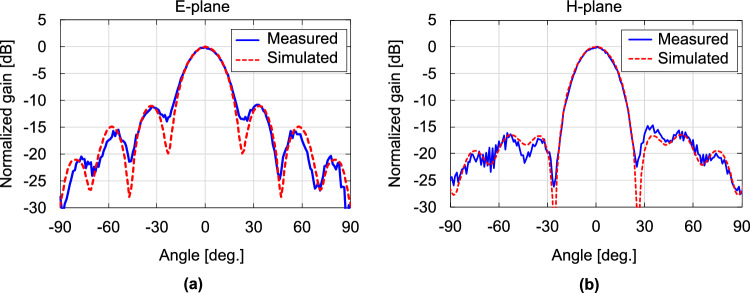
Measured and simulated radiation patterns of DCA3, (**a**) E-plane, (**b**) H-plane.

We measured the antenna characteristics by inserting the DCA directly into the OEWG (Fig. 12b). We used a commercially available near-field probe (Elmika AOEWGP-010E/1) as an OEWG. The near-field probe used here had a tapered structure to reduce the scattering. We have confirmed that the radiation patterns are consistent with the tapered flangeless and flangeless straight waveguides. The antenna gain was measured by the gain comparison method using a signal generator (Keysight E8257D), a spectrum analyzer (Keysight N9041B), and WR-3.4 frequency extenders (Virginia Diodes SGX 638 and SAX 754). Figure 13 shows the frequency response of the measured antenna gain. Except the small ripple in the high-frequency range, the characteristics generally agreed with the simulation results. The ripple in the measurement results may have been due to multiple reflections between the Tx and Rx of the gain measurement setup. Although the volume of DCA1 was approximately $13\%$ smaller than that of the DCA with the waveguide flange in^32^, the antenna gain of DCA1 at 300 GHz was 14.6 dBi, which was almost the same as that of the DCA with the waveguide flange. Although DCA2 had the same aperture area as the DCA with the waveguide flange in^32^, its antenna gain was 16.1 dBi, which was 1.1 dB higher than that of the DCA with a waveguide flange; this antenna gain was obtained at 300 GHz by increasing *b* from 1.36 to 1.70 mm. DCA3 had an antenna gain of 17.2 dBi at 300 GHz, and the 3-dB bandwidth covered the entire WR-3.4 waveguide bandwidth. The aperture efficiency $\eta _{A}$ is defined as

1 $$\begin{aligned} \eta _{A} = \frac{\lambda ^{2}}{4\pi A}G \end{aligned}$$

where $\lambda$ is the wavelength of the electromagnetic wave, *A* is the physical aperture area of the antenna, and *G* is the antenna gain. DCA3 had a high aperture efficiency of $185\%$ at 300 GHz because the dielectric-side surface contributed to the radiation. Figure 14 shows the measured $\vert \hbox {S}_{11}\vert$, which were obtained using a vector network analyzer (Keysight PNA-X N5247B) and WR-3.4 frequency extenders (Virginia Diodes VNAX 643 and VNAX 644). All DCAs were experimentally confirmed to operate with $\vert \hbox {S}_{11}\vert$ below $-10$ dB throughout the WR-3.4 waveguide bandwidth. The maximum value of $\vert \hbox {S}_{11}\vert$ experimentally obtained for DCA3 is $-15.4$ dB, which satisfies VSWR $< 1.5$ ($\vert \hbox {S}_{11}\vert$ $< -14.0$ dB). Figure 15 shows the measured and simulated radiation patterns of DCA3 at 300 GHz. The experimentally obtained E- and H-plane patterns agreed well with the simulations, including their main-lobe shapes, side-lobe positions, and SLLs.

## Conclusions

We designed a DCA connected to a WR-3.4 OEWG and performed simulations and experiments to confirm their characteristics in the 300 GHz band. For a given DCA aperture dimension, a specific DCA length achieved the maximum antenna gain, a main lobe with good rotational symmetry, and low SLLs. Based on these findings, a DCA prototype with an antenna aperture area of 1.5 mm $\times$ 1.5 mm and an antenna length of 2.35 mm was fabricated. The fabricated DCA exhibited WR-3.4 full-band operation (220–330 GHz) with $\vert \hbox {S}_{11}\vert$ of less than $-15.4$ dB, which satisfies VSWR $< 1.5$. The experimentally obtained antenna gain was 17.2 dBi at 300 GHz. The proposed antenna can be applied to other THz frequencies through wavelength normalization while considering the material parameters of the target frequency. Therefore, DCA without a metal plate is a promising antenna for future transceiver chips and mobile devices used in high-speed, high-capacity terahertz wireless communication systems.

## Data Availability

The original contributions presented in the study are included in the article; further inquiries can be directed to the corresponding author.
